# A comparative study on the prediction of the BP artificial neural network model and the ARIMA model in the incidence of AIDS

**DOI:** 10.1186/s12911-020-01157-3

**Published:** 2020-07-02

**Authors:** Zeming Li, Yanning Li

**Affiliations:** 1 School of Public Health of Peking University, NO.38 Xueyuan Road, Beijing, 100191 China; 2 School of Public Health Guangxi Medical University, NO.22 Shuangyong Road, Nanning, 530021 China

**Keywords:** AIDS, Prediction, BP artificial neural network model, ARIMA model

## Abstract

**Background:**

As a kind of widely distributed disease in China, acquired immune deficiency syndrome (AIDS) has been quickly growing each year, become a serious problem and caused serious damage to the life and health of people and the social events of China and the world because of its high fatality rate. It has been much concerned by all aspects of society. Therefore, developing early warning technology and finding the trend of early development are of quite significance to prevent and control human immunodeficiency virus (HIV)/AIDS. This study aimed to explore a suitable model for the morbidity of AIDS in China and establish a professional and feasible disease prediction model for the prevention and control works of AIDS.

**Methods:**

At present, the traditional linear model is still utilized by most scholars to predict the incidence of HIV/AIDS. In addition, some scholars may attempt to use the nonlinear prediction model. Both prediction models showed good fitting and prediction effects. In China, the incidence of AIDS presents linear and nonlinear characteristics. In this research, the nonlinear back propagation artificial neural network (BP-ANN) model and the typical auto-regressive integrated moving average (ARIMA) linear model were applied to predict the incidence of HIV/AIDS and compare their fitting effects.

**Results:**

Both models were capable of predicting the expected cases of AIDS. It was seen that ARIMA and BP-ANN models could be used to forecast the monthly incidence of HIV/AIDS, but the fitting and forecasting effects of the nonlinear BP neural network model were better than those of the traditional linear ARIMA model.

**Conclusions:**

In summary, it was further concluded that the BP-ANN model was a suitable way to monitor and predict the change trend and morbidity of AIDS in China.

## Background

Human Immunodeficiency Virus (HIV) is a deadly virus weakening and attacking the immunity system, which can induce Acquired Immune Deficiency Syndrome (AIDS) that is recognized as one of notifiable communicable diseases around the world [1]. During the last decades, AIDS has been seen as an epidemic that becomes a serious public health problem and social event all over the world, causes serious damage to the life and health of people and affects all aspects of society. In the global context, 36.9 million people were carried with HIV, and 0.94 million people died of HIV-associated diseases by the end of 2017 [2]. Since 1998, the number of provinces affected by HIV/AIDS has reached 31, which still sees a rapid increase in China [3]. The epidemic of AIDS/HIV has been worsened to pose serious threats to public health. Each year, it seems that new infection cases are increasing in China [4, 5]. In 2015, about 571,000 people (15 years old and above) were infected with HIV [6].

Therefore, it is a must to prevent and control the prevalence of AIDS in China. A number of policies on the prevention and control works of HIV disease have been issued by the government. In order to supervise the spread of HIV/AIDS, the National Notifiable Disease Surveillance System was organized in 1995, and the surveillance data for primarily affected populations was collected [7, 8]. Since 2004, this system has been applied to monitor the prevalence of HIV and HIV-related behaviors [9].

Over the past few years, mathematical models have been used to successfully predict the incidence of HIV/AIDS. In the 1980s, the model suggested by the Joint United Nations Programme on HIV/AIDS (UNAIDS) was adopted to forecast HIV-infected patients in many countries so as to identify the growing trend of the disease. The methods are the Workbook Method [10], Estimation and Projection Package (EPP) method [11], Spectrum AIDS Impact Model [12] as well as Asian Epidemic Model (AEM) [13]. Due to the changing incidence of AIDS, it is necessary to think through its influence factors. In these models, adequate indicators are required to fit in different estimation and prediction curves about the epidemic situation of HIV/AIDS. Otherwise, the results will greatly deviate from the actual situation. The features of four models are as follows, Workbook, the parameters required are some relatively fixed demographic indicators, including local adult population, gender composition, base of various high-risk groups and high and low values of infection rates, base of various low-risk groups and high and low values of infection rates, etc. [10]. Spectrum AIDS Impact Model, HIV-infected people receive Antiretroviral Therapy (ART) to extend their survival time. The change in survival time will affect the prediction results of SPECTRUM [14]. EPP, the number of people receiving treatment has increased with the promotion and use of condoms. The improvement of treatment methods and other prevention and control works have reduced the quality and representativeness of monitored data, which exerts a direct influence on EPP’s estimation and prediction of epidemic situations [15]. AEM, its monitoring indicators have a large number of difficult items. Monitoring data has high-quality requirements. Only on the premise of sufficient data and quality assurance can appropriate model parameters be obtained. Then, predictions can be made. Otherwise, major mistakes are easy to make [16].

Also known as the historical extension prediction method, the time series prediction method is a kind of historical data extension prediction that is a method of extrapolating and predicting the development trend of things, which can be reflected by time series. More common traditional time series prediction methods include the Auto-Regressive Integrated Moving Average (ARIMA) model, exponential smoothing method, etc., among which ARIMA is the most representative. Considered as one of the major ways to make time series analysis, the ARIMA model involves the changes of trends, random interference and periodic variations and the invariance of other related random variables during time series analysis. Earnest et al. believed that the ARIMA model was quite easy and fast to set related parameters on the prediction of communicable diseases [17]. The establishment of the ARIMA model requires collecting relevant historical data, processing data in advance according to its stability requirements, drawing the diagram of autocorrelation coefficients and partial correlation coefficients to determine the optimal model and finally use it to predict the development trend. Nowadays, ARIMA is used to estimate the mortality of influenza, malaria and other infectious diseases.

In most cases, nonlinear structures are adopted during time series analysis as adequate results cannot be obtained from linear models. In many domains, the Artificial Neural Network (ANN) is applied due to its possibility of getting over the limitations of linear models [18] and analyzing the strongly-coupled and highly-nonlinear correlations between multiple input and output variables. In nonlinear artificial neural network models, particularly the Back Propagation Artificial Neural Network (BP-ANN), the BP-ANN model can improve prediction accuracy close to various functions of arbitrary nonlinear structures [19], and accommodate more multidimensional inputs to improve the accuracy of predictions because of its inherent self-learning property, simple structure and strong simulation ability.

The data of AIDS incidence in China has shown a coexistence of linearity and nonlinearity. In this paper, it was suggested that the nonlinear relationships should exist for the monthly morbidity of AIDS while accuracy relations should not be extracted from the linear model. Two models, namely ARIMA and BP-ANN, were established to forecast the morbidity of HIV/AIDS during the period of 2007–2016. By comparison, the future growing trend of HIV/AIDS was described for early detection and warning.

## Methods

### ARIMA model

As a common linear model in time series analysis, the ARIMA model is usually constructed as ARIMA (p, d, q) (P, D, Q) _S_, p, d, q, P, D, Q and S refer to autoregressive order, number of difference, moving average order, seasonal autoregressive order, number of seasonal difference, seasonal moving average order and time-series of cyclical pattern respectively. Graphs of Auto-Correlation Function (ACF) and Partial Auto-Correlation Function (PACF) were utilized to determine the ARIMA model [20]. The construction of an optimal model needed to think about minimum Bayesian Information Criterions (BIC) and stable multi-correlation coefficient, statistically significant parameter estimates and residuals as white noise. The ARIMA model was constructed through former forecasting errors and past series values, and developed according to the following procedures: Diagnostic checking, estimation and identification. During the identification process, the ACF and PACF of transformed information would determine seasonal and non-seasonal orders. Conditional least-squares modes were used to estimate parameters. During the diagnosis process, white noise tests were conducted to verify the adequacy of the model in the series and check whether residuals were independently and positively distributed. In this way, a few ARIMA models would be possibly identified [21]. Finally, a suitable model would be selected to forecast morbidity.

### BP-ANN model

As one of artificial intelligence (AI) technologies, ANN has been generally applied to fit in nonlinear models with the capability of recognizing the principles of accurate forecasting and offering help to make decisions [22]. A large number of connected nonlinear units are contained in the ANN model for data storage self-learning process [23]. Among ANN models, the BP-ANN model is a type of multi-layered feed forward neural network.

As a system with learning ability, ANN can develop knowledge so as to exceed the original knowledge level of designers. Its learning and training methods can be divided into two types: One is supervised or tutored learning in which given sample criteria are used for classification or imitation; the other is unsupervised or untutored learning in which only learning styles or certain rules are set and the specific learning content varies with the environment (namely the situation of input signal) of the system that can automatically find the characteristics and regularity of the environment.

ANN is an implicit mathematical processing method and a typical black-box modeling tool. In general, it is only necessary to give the input and output data of the modeling object instead of knowing its structure, parameters and dynamic characteristics. Through the training of information samples, the neural network can have the brain’s ability of memory and recognition. Without any prior formulas or modeling, the ANN can self-learn, obtain the mapping relationship between input and output from existing data, store the mapping relationship in each neuron in the form of multigroup weights and thresholds to constitute network knowledge, and use it to predict similar factors. Neural network models are widely used in signal processing, pattern recognition, control, analysis and prediction and other aspects because of their nonlinear characteristics, numerous parallel distribution structures as well as learning and inductive ability.

**Fig. 1:**
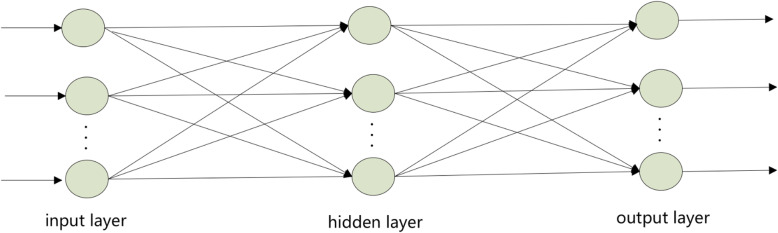
BP-ANN classic structure diagram

Three layers of the BP-ANN model architecture were contained in the paper, which including the input layer, the hidden layer, and the output layer. Each layer has at least one neuron, which connects to neurons in different layers. The classic structure is shown in Fig. 1. This structure is simple, clarity and can enable each neuron to establish a suitable linear or non-linear relationship between input and output, while without limiting in output between − 1 and 1. The core of BP-ANN is each neuron in the input layer as an independent variable; the hidden layer is responsible for internal operations (imitating the human brain), especially non-linear operations; each neuron in the output layer represents a dependent variable. The calculation of BP-ANN is to find the minimum value of the error function.

### Model validation and statistical comparisons

Based on the same training set, ARIMA and BP-ANN models were subsequently established to forecast exclusively experimental information. The validity of these models was evaluated by cross validation. Mean Absolute Error (MAE), Mean Square Error (MSE) and Mean Absolute Percentage Error (MAPE) were used to make a statistical comparison of forecast and real morbidity.

### Information analysis based on computer software

The ARIMA model was analyzed by using software SPSS26 and Eviews6.0. Neural Network Toolbox in Matlab 2019 was used to evaluate the BP-ANN model. All the analysis results showed significant differences, namely *P* < 0.05.

### Data sources

According to the report on statutory infectious diseases in China, the monthly data about China’s AIDS cases reported from January 2004 to December 2016 was collected as the original data to establish the models, to predict the incidence of AIDS in 2017. Compare forecast incidence of AIDS and actual incidence of 2017, to verify the model fitting effect.

In ARIMA model, The monthly incidence of AIDS in China from 2004 to 2016 was modeled, and predicting the monthly incidence in 2017. The actual value of monthly incidence in 2017 was used as a reference to verify the model. In BP-ANN model, the set of information was classified into three subsets, namely training, validation and test sets. In the training set, the incidence data of the past three years was used to predict the incidence of the fourth year in validation set. The incidence rate in January of t_1_-t_3_ years was used to estimate that in January of the t_4_ year; the incidence rate in February of t_1_-t_3_ years was used to estimate that in February of the t_4_ year, and so on. Then, the incidence rate in the same month of t_2_-t_4_ years was used to predict that in the same month of the t_5_ year, the same month of t_3_-t_5_ years was used to predict the incidence rate of the same month of the t_6_ year, in turns. Finally, the data of 2017 was selected as the test set to verify network performance. All incidence data were numbered in chronological order, with P1, P2 and P13 representing respectively the incidence data in January 2004, February 2004 and January 2005, and so on. The specific data diversity is presented in the following Table 1. Such data diversity method could be adopted to better learn and train network models, and avoid overlearning and overfitting [24].

**Table 1:** Three date set in BP-ANN

No.	training set			validation set
1	P1(2004–01)	P13(2005–01)	P25(2006–01)	P37(2007–01)
2	P2(2004–02)	P14(2005–02)	P26(2006–02)	P38(2007–02)
3	P3(2004–03)	P15(2005–03)	P27(2006–03)	P39(2007–03)
i	P(i)	P(i + 12)	P(i + 24)	P(i + 36)
82	P82(2010–10)	P94(2011–10)	P106(2012–10)	P118(2013–10)
83	P83(2010–11)	P95(2011–11)	P107(2012–11)	P119(2013–11)
84	P84(2010–12)	P96(2011–12)	P108(2012–12)	P120(2013–12)
85	P85(2011–01)	P97(2012–01)	P109(2013–01)	P121(2014–01)
109	P109(2013–01)	P121(2014–01)	P133(2015–01)	P145(2016–01)
119	P119(2013–11)	P131(2014–11)	P143(2015–11)	P155(2016–11)
120	P120(2013–12)	P132(2014–12)	P144(2015–12)	P156(2016–12)
121	P121(2014–01)	P133(2015–01)	P145(2016–01)	P157(2017–01)
131	P131(2014–11)	P143(2015–11)	P155(2016–11)	P167(2017–11)
132	P132(2014–12)	P144(2015–12)	P156(2016–12)	

## Results

### Features of time series analysis in the report rate of AIDS

**Fig 2:**
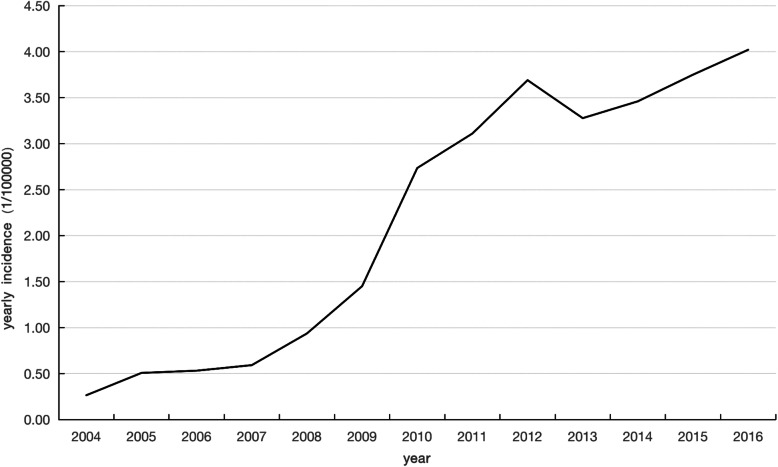
The yearly incidence of AIDS/HIV in China from 2004 to 2016

**Fig. 3:**
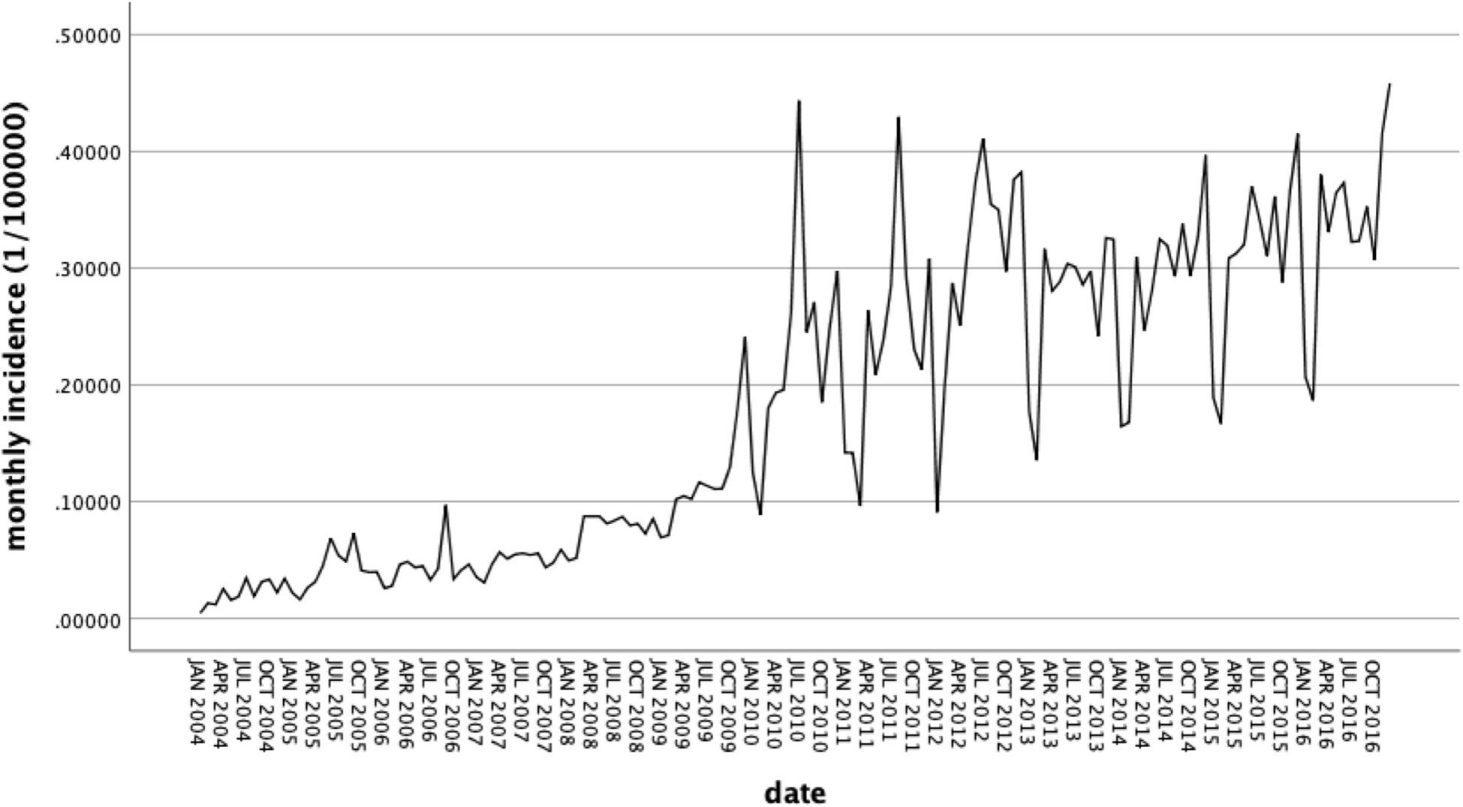
Chinese AIDS monthly incidence from 2004 to 2016

According to the surveillance data from January 2004 to December 2016, the figure of monthly incidence rates showed a trend of sharp increase from 2010 to 2016 the peak incidence existed in 2012 (Fig. 2). Table 2 showed the average of monthly morbidity of AIDS at the period between 2004 and 2016. The annual incidence rate was between 0.2648 and 4.0211 per 100,000 people from 2004 to 2016. Figure 3 shown that the monthly incidence of AIDS in China was cyclical. The lowest point was generally between January and February of each year, and the highest point was generally from July to December of each year. In summary, the monthly incidence of AIDS in China during the 13-year period from 2004 to 2016 had been cyclical and increasing year by year.

**Table 2:** The average of yearly Incidence and growth rate of HIV/AIDS in China, 2004–2016

year	Incidence (per 100,000 people)	chain growth rate(%)	growth rate(%)
2004	0.2648	–	–
2005	0.5076	91.6994	91.6994
2006	0.5320	4.7930	100.8875
2007	0.5921	11.3056	123.5989
2008	0.9368	58.2124	253.7613
2009	1.4507	54.8668	447.8588
2010	2.7356	88.5664	933.0778
2011	3.1107	13.7129	1074.7432
2012	3.6908	18.6491	1293.8218
2013	3.2777	−11.1931	1137.8097
2014	3.4608	5.5865	1206.9600
2015	3.7506	8.3738	1316.3897
2016	4.0211	7.2122	1418.5423

### ARIMA model

#### Model identification

**Fig. 4:**
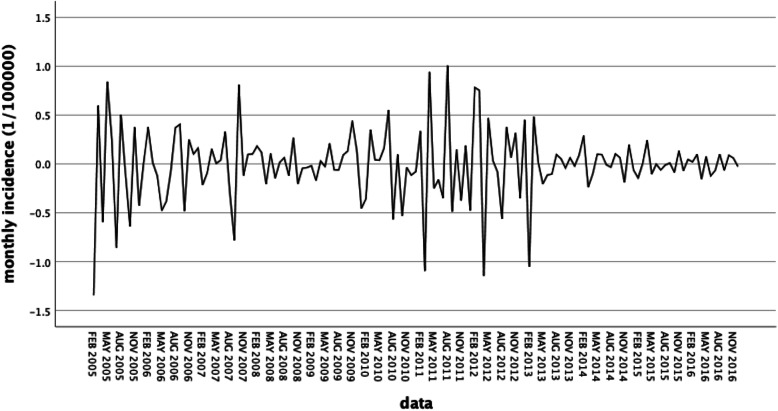
AIDS monthly incidence transformation: natural logarithm, difference, seasonal difference

The time series from January 2004 to December 2016 were used to establish the model for the morbidity of AIDS, which were not stationary owing to seasonality. After the natural logarithmic transformation was performed, one general difference, one seasonal difference, time plots after transformation are shown in Fig. 4. Transformed time series appeared to be quite stationary.

**Fig. 5:**
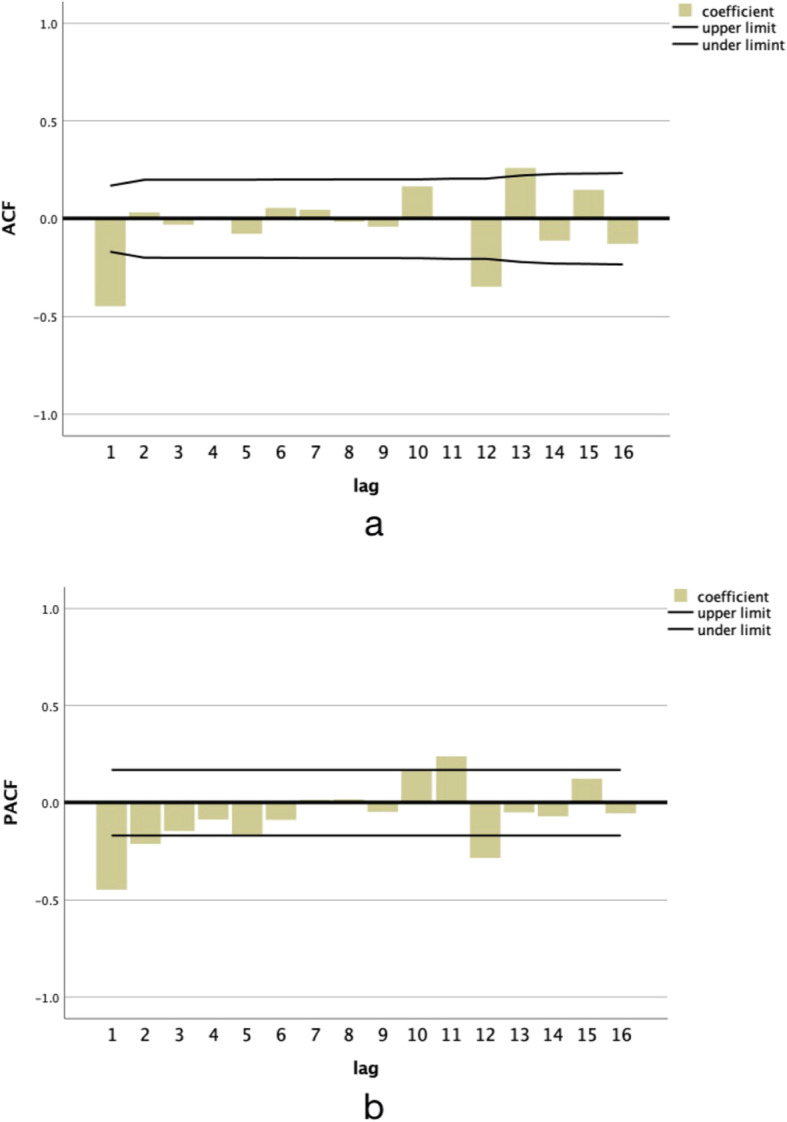
ACF and PACF graphs of AIDS monthly incidence

ACF and PACF were used to describe the characteristics of series, select models and determine the order of key points. ACF was utilized to explain the correlation of several adjacent data as the coefficient of the relationship between series and their own historical or stagnant series. When the lag = 1, it is the first-order autocorrelation coefficient (*p* = 1), which indicates that there is a correlation between adjoining points; lag = 2 means the second-order autocorrelation coefficient (*p* = 2), which indicates that two adjoining points are also closely related, but generally the autocorrelation coefficient in ACF does not exceed 2. The ACF in Fig. 5-a shows that the autoregressive value broke through the wireframe of confidence interval only when lag = 1, indicating that the series had a high correlation within the first order. PACF was to test whether the partial correlation coefficient of each order was statistically significant one by one from higher to lower order until the first one was significant. The order of coefficients of PACF determines the highest order in the model. As shown in the PACF diagram (Fig. 5-b), the partial regression coefficient exceeded the confidence interval when lag = 1 and 2, indicating the feasibility of modeling within two orders. Therefore, this study considered that the partial regression coefficient decrease sharply after lag = 1, so neglected lag = 2.

As displayed from Fig. 5a and b, the model was initially determined as ARIMA(p, d, q) × (P, D, Q) s (General Multiplicative Seasonal Model). Since one general difference (d = 1) and one seasonal difference (D = 1) were performed in data pre-processing, ARIMA(p,1,q) × (p,1,q) _12_ models with all order combinations for all autocorrelation delay coefficients *p* ≤ 1(*P* ≤ 1) and moving average delay coefficients q ≤ 1(Q ≤ 1) were selected as primary models.

All primary models were used to simulate and model the monthly incidence of AIDS. The statistics, BIC and parameter estimates of the models obtained are shown in Table 3. The table selected stationary R-squared and BIC with the relatively smallest value, and the model whose residual was white noise was the optimal one. According to the minimum BIC = -6.091 and white noise test for residual errors, Ljung-Box Q [18] =13.909, *P* > 0.05, which indicated that goodness-of-fit considered ARIMA (0,1,1) × (0,1,1)_12_ as the most suitable model.

**Table 3:** Parameter estimation and model verification of ARIMA model

Models	Fitted Model Statistics					Ljung-Box Q(18)	
	Stationary R^**2**^	RMSE	MAPE	MAE	BIC	Statistics	Sig.
ARIMA(0,1,0) × (0,1,0)_12_	0.000	0.087	30.213	0.047	−4.848	78.375	0.000
ARIMA(0,1,0) × (0,1,1)_12_	0.205	0.057	26.869	0.037	−5.668	48.93	0.000
ARIMA(0,1,0) × (1,1,0)_12_	0.115	0.066	28.243	0.041	−5.361	53.683	0.000
ARIMA(0,1,0) × (1,1,1)_12_	0.210	0.057	26.806	0.037	−5.609	46.879	0.000
ARIMA(0,1,1) × (0,1,0)_12_	0.274	0.061	24.461	0.036	−5.522	30.871	0.021
**ARIMA(0,1,1) × (0,1,1)**_**12**_	**0.419**	**0.045**	**22.464**	**0.030**	**−6.091**	**13.909**	**0.605**
ARIMA(0,1,1) × (1,1,0)_12_	0.365	0.051	23.118	0.033	−5.834	13.873	0.608
ARIMA(0,1,1) × (1,1,1)_12_	0.428	0.046	22.079	0.030	−6.032	10.764	0.769
ARIMA(1,1,0) × (0,1,0)_12_	0.197	0.068	26.551	0.040	−5.307	53.543	0.000
ARIMA(1,1,0) × (0,1,1)_12_	0.369	0.049	23.588	0.033	−5.927	16.727	0.403
ARIMA(1,1,0) × (1,1,0)_12_	0.305	0.056	24.379	0.036	−5.665	19.492	0.244
ARIMA(1,1,0) × (1,1,1)_12_	0.374	0.049	23.353	0.033	−5.874	16.066	0.378
ARIMA (1,1,1)×(0,1,0)_12_	0.274	0.061	24.485	0.036	−5.479	30.781	0.014
ARIMA (1,1,1)×(0,1,1)_12_	0.420	0.045	22.494	0.030	−6.049	13.949	0.529
ARIMA (1,1,1)×(1,1,0)_12_	0.365	0.052	23.095	0.033	−5.790	13.923	0.531
ARIMA (1,1,1)×(1,1,1)_12_	0.428	0.046	22.081	0.030	−5.990	10.758	0.705

#### Forecast analysis with ARIMA

**Fig. 6:**
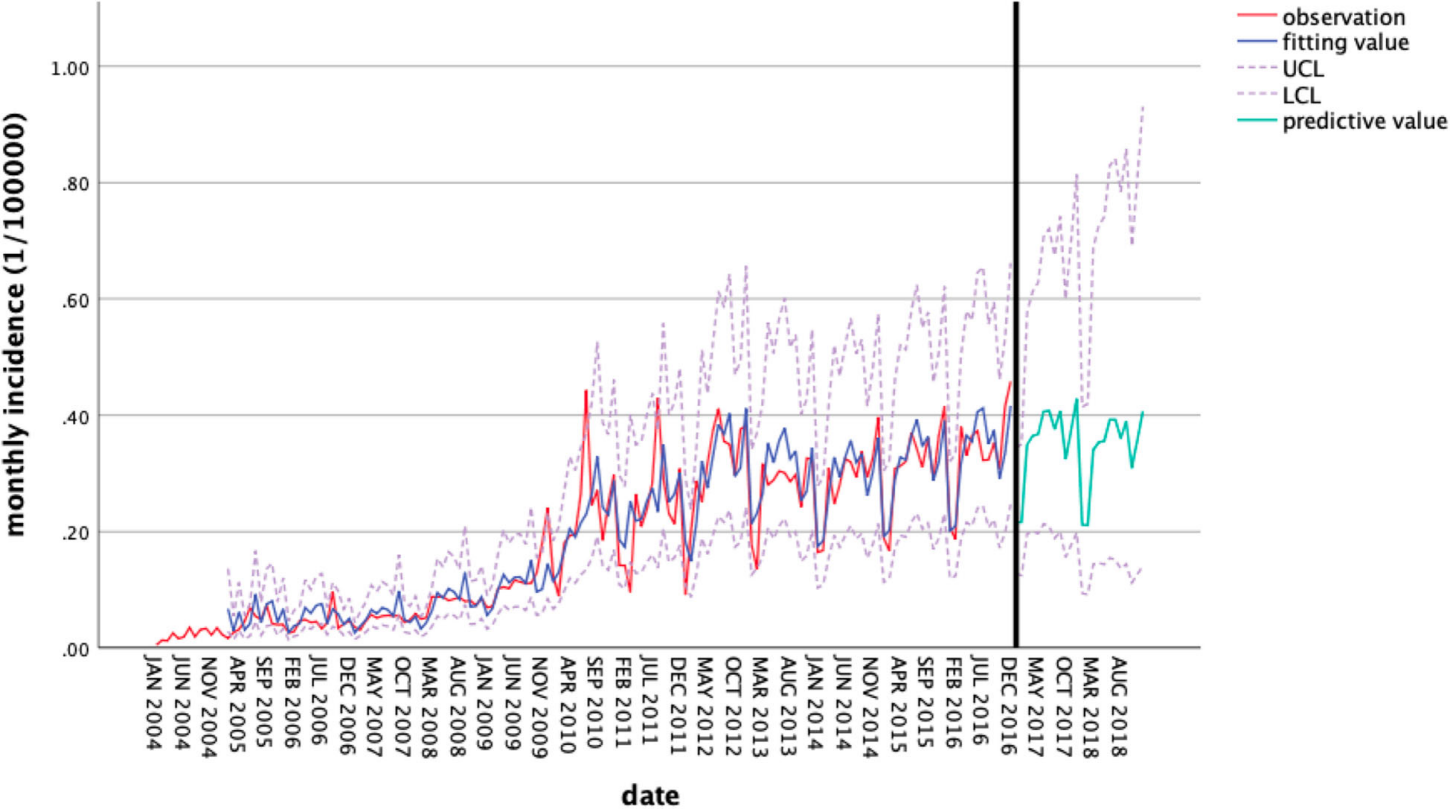
Comparison of ARIMA model prediction and the actual incidence

ARIMA(0,1,1) × (0,1,1)_12_ was used to predict the monthly incidence of ADIS from January to December 2017. The results are shown in Table 4. It can be seen from Fig. 6 that the change trend of monthly incidence fitted by this model was basically consistent with original data, and the fitting effect was satisfactory. With the extension of prediction time, 95% confidence interval of predicted value would widen and the accuracy of predictions saw a gradual decline, which was consistent with the conclusion of Xiao-Mei M [25] and Li-Ping R [26].

**Table 4:** The predictive monthly incidence of AIDS in 2017 based by ARIMA(0,1,1) × (0,1,1)_12_

Month	Actual value	Predictive value	UCL	LCL
201,701	0.1810	0.2164	0.3437	0.1280
201,702	0.2405	0.2162	0.3502	0.1246
201,703	0.3746	0.3496	0.5772	0.1966
201,704	0.2994	0.3645	0.6128	0.2002
201,705	0.3634	0.3672	0.6281	0.1970
201,706	0.4279	0.4065	0.7069	0.2132
201,707	0.358	0.4077	0.7204	0.2092
201,708	0.3905	0.3756	0.6740	0.1887
201,709	0.3821	0.4073	0.7418	0.200
201,710	0.3244	0.3241	0.5988	0.1563
201,711	0.4438	0.3752	0.7031	0.1773
201,712	0.4789	0.4284	0.8137	0.1986

### BP-ANN model

The set of information was divided into training, test and validation data sets in the ARIMA model. The BP-ANN model was established by Matlab 2019 to predict the incidence of HIV/AIDS in China in 2017.

#### Network architecture

The BP-ANN modeling process has the following three steps:

1) original data was divided into three data sets, namely training, validation and test sets. The training set was used to train models and select the optimal network; the verification set was utilized to monitor the entire network training process; the test set was applied to verify the performance of the selected optimal network model. In network training, training and validation sets are usually selected to enter the network alternately in order to avoid overfitting, which means that established network models explain not only the variation of the observed population but also the fluctuations and errors of individual samples in the population [24].

2) After centralized training, repeated learning, forward and backward propagation of information, and continuous adjustment of network weights, the mean square error (MSE) of validation set would be minimized or reach a predetermined number of iterations [27, 28].

3) As a set of data coming from the same whole with training and verification sets and failing to enter network training, the test set can be used to evaluate established network models to obtain objective and extrapolative effective results.

In the training set of this model, the incidence data of the past three years was used to learn the incidence of the fourth year, such as the incidence rate in January of t_1_-t_3_ years was used to estimate that in January of the t_4_ year, then the incidence rate in the same month of t_2_-t_4_ years was used to predict that in the same month of the t_5_ year, in turn. With such data diversity method could be adopted to better learn and train network models, and avoid overlearning and overfitting.

After dividing data into three sets, network parameters are set up, such as number of network layers, nodes and iterations, the allowable error, and the learning algorithm used.

After the data set has been partitioned, the number of network layers, number of neural nodes, number of iterations, allowable error, learning algorithms and other network parameters of the model should be set before starting training.

To determine the number of network layers. A study by Robert Hecht-Nielsen in 1989 has shown that the feedforward network of a hidden layer can map continuous functions within all closed intervals [29]. A three-layer BP network model can complete any mapping from n to m dimensions. More than two hidden layers should only be considered when learning discontinuous functions. As long as the number of nodes in the hidden layer can be reasonably selected, the BP network model of a hidden layer has also strong nonlinear mapping capability, fast training speed, and good convergence ability. Hence, a three-layer BP network model was selected and a hidden layer was adopted in this study.

To determine the number of neurons (also called nodes) in each layer. The number of nodes in the input layer and the output layer is generally determined according to the data characteristics of the study. In this study, according to the data diversity and the predicted monthly incidence rate, the number of nodes in the input layer is 3, and the number of nodes in the output layer is 1. The number of nodes in the hidden layer has a certain influence on the performance of the neural network model. Too few neural nodes will cause small learning capacity, and failure to completely learn samples and laws of sample storage; Too many neural nodes will cause the network to be bloated, so that the learning speed may slow down and the irregular parts (such as white noise) of sample data may be stored into the network, resulting in poor network performance and generalization ability. At present, the number of nodes in the BP-ANN hidden layer is almost calculated and estimated by the empirical formula. Based on the literature review, this study uses four formulas and two empirical formulas that are the most commonly used to infer the approximate number of neural nodes in the hidden layer, and the formulas are as follows:

$$ \left(\mathrm{Kuarycki}\right)\mathrm{m}=3\mathrm{N} $$

$$ (Maren)\mathrm{m}=\mathrm{M}\left(\mathrm{N}+1\right) $$

$$ Lippmann:m=\sqrt{M}\times N $$

$$ \left( Hecht- Nielsen\right)\mathrm{m}=2\mathrm{N}+1 $$

$$ {\displaystyle \begin{array}{l} Empirical\ Formulas:m=\sqrt{M\times N}+a\\ {}m={\log}_2M\end{array}} $$

where M represents the number of input layer nodes; N represents the number of output layer nodes; “m” represents the number of hidden layer nodes; “a” is the regulation constant with values between 1 and 10. In this study, the number of nodes in hidden layer ranges from 3 to 12.

Select the learning algorithms and structures, initialize the model. Matlab provides 10 (a total of 11) BP neural network model learning algorithms, including Levenberg-Marquardt algorithm (Train-lm), One Step Secant (OSS) algorithm (Trainoss), conjugate direction algorithm (T-trainscg), Polak-Ribiere algorithm (Traincgp), Fletcher-Reeves algorithm (Traincgf), resilient BP algorithm (Trainrp), self-adaptive learning rate algorithm (Traingda and Traingdx), gradient descent with momentum (Traingdm) and batch gradient descent training function (Traingd). Among them, the Levenberg-Marquardt algorithm, the L-M algorithm for short, is the most widely used nonlinear least square algorithm at present because of its fast convergence speed.

In this study, three years of data were randomly selected from the data set of the monthly incidence of AIDS. After the normalization of data by the PRESTD function, estimated from 3 to 12 nodes in the hidden layer and above 11 algorithms were used to combine into the neural network models of 110 structures. Small sample data was input, and each structure was iterated 20 times to calculate their MSEs respectively. The smaller MSE was, the better the fitting effect of the network model would be and the closer the neural network prediction would be to the real value. The combination of the structure and algorithm of the minimum MSE was shown in Table 5. The combined BP neural network model with the minimum MSE = 0.001863 was the optimal model, and the optimal learning algorithm was the L-M algorithm.

**Table 5:** MSE of 11 BP-ANN algorithms base on 3–12 neurons in the hidden layer

Algorithm	Number of neurons in the hidden layer									
	3	4	5	6	7	8	9	10	11	12
Traingd	0.597710	0.633182	0.566311	0.888439	0.778596	0.895304	1.025611	1.057920	0.425543	0.382488
Traingdm	0.003257	0.002775	0.003120	0.003124	0.003389	0.003088	0.003015	0.003237	0.003293	0.003116
Traingda	0.002978	0.002820	0.003169	0.002910	0.002736	0.003304	0.002894	0.003054	0.003250	0.002987
Traingdx	0.004025	0.003410	0.003930	0.003967	0.003496	0.002735	0.003296	0.003464	0.003186	0.003055
Trainrp	0.004357	0.004044	0.004410	0.004013	0.004315	0.004017	0.004347	0.004304	0.004002	0.003873
Traincgf	0.004123	0.004409	0.003290	0.003908	0.003490	0.004200	0.004084	0.003001	0.004252	0.004482
Traincgp	0.003626	0.004292	0.003758	0.002979	0.003060	0.003433	0.004048	0.004186	0.004122	0.003273
Traincgb	0.003661	0.002862	0.002901	0.002945	0.003922	0.003591	0.003041	0.003591	0.002966	0.002799
Trainscg	0.004381	0.004148	0.004444	0.004257	0.004166	0.004352	0.004403	0.004491	0.003700	0.004392
Trainoss	0.003074	0.003489	0.002980	0.002927	0.003281	0.002651	0.002948	0.003391	0.003032	0.003330
Trainlm	0.002369	0.002293	0.002123	**0.001863**	0.002042	0.002313	0.002330	0.002365	0.002445	0.002491

#### Forecast analysis with BP-ANN

**Fig. 7:**
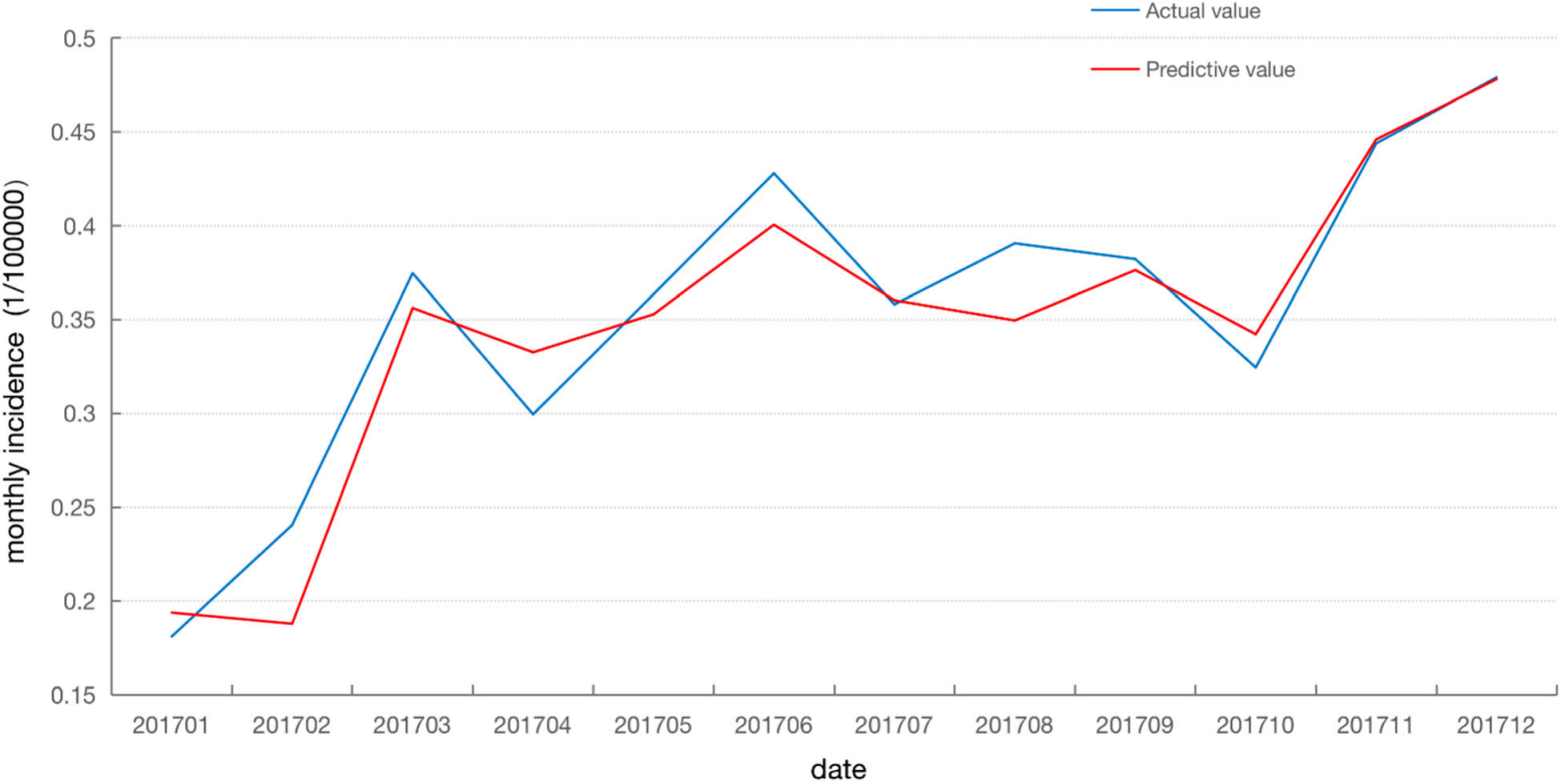
Comparison of BP-ANN model prediction and the actual incidence

The BP neural network fitting curve for the incidence of HIV/AIDS in 2017 was obtained by inputting the test set into the trained BP-ANN and using the stored black box to operate network models (Fig. 7 and Table 6 of fitted value). By comparing the predicted value with the actual incidence, the fitted value of the BP-ANN model was very close to the actual monthly incidence of AIDS.

**Table 6:** The predictive monthly incidence of AIDS in 2017 based by BP-ANN

Month	Actual value	Predictive value
201701	0.1810	0.193743
201702	0.2405	0.187785
201703	0.3746	0.356085
201704	0.2994	0.332513
201705	0.3634	0.352712
201706	0.4279	0.400424
201707	0.3580	0.360190
201708	0.3905	0.349451
201709	0.3821	0.376242
201710	0.3244	0.342154
201711	0.4438	0.445962
201712	0.4789	0.477938

### Comparative analysis

This study mainly compared and evaluated the prediction effects of the ARIMA time series model and BP-ANN model of the following three error evaluation indicators. In Table 7, the observed values were compared with the predicted ones in a point-to-point manner. The modeled MSE, MAE and MAPE in the ARIMA model were 0.0020, 0.0301 and 22.4638 respectively. However, three residuals in the BP-ANN model were 0.0019, 0.0129 and 1.2139 respectively.

**Table 7:** Comparison of the fitting and prediction performance of the two models

Prediction error	ARIMA	BP-ANN
MSE	0.0020	0.0019
MAE	0.0301	0.0129
MAPE	22.4638	1.2139

When the morbidity of HIV/AIDS from 2004 to 2016 was set as the original data, models were established to forecast the morbidity of AIDS in 2017. The predicted incidence of AIDS was compared with the actual incidence of AIDS in 2017 so as to verify the fitting effects of models. Ultimately, the ARIMA (0,1,1) (0,1,1)_12_ structure was considered to be the most suitable time series model with white noise testing LB [18] = 13.909, *P* > 0.05, which meant that the model was effective. In the model, error parameters were MSE = 0.0020, MAE = 0.0301 and MAPE = 22.4638. The selected BP neural network model was seen as the optimal one with the L-M algorithm. In the model, MSE iterated 16 times was 0.0019, MAE was 0.0129 and MAPE was 1.2139. The fitting error of the BP-ANN model was significantly smaller than that of the ARIMA model while its forecasting accuracy was higher than that of the ARIMA model [30–32]. It was seen that the BP-ANN model was more effective in predicting the morbidity of AIDS in China.

**Fig. 8:**
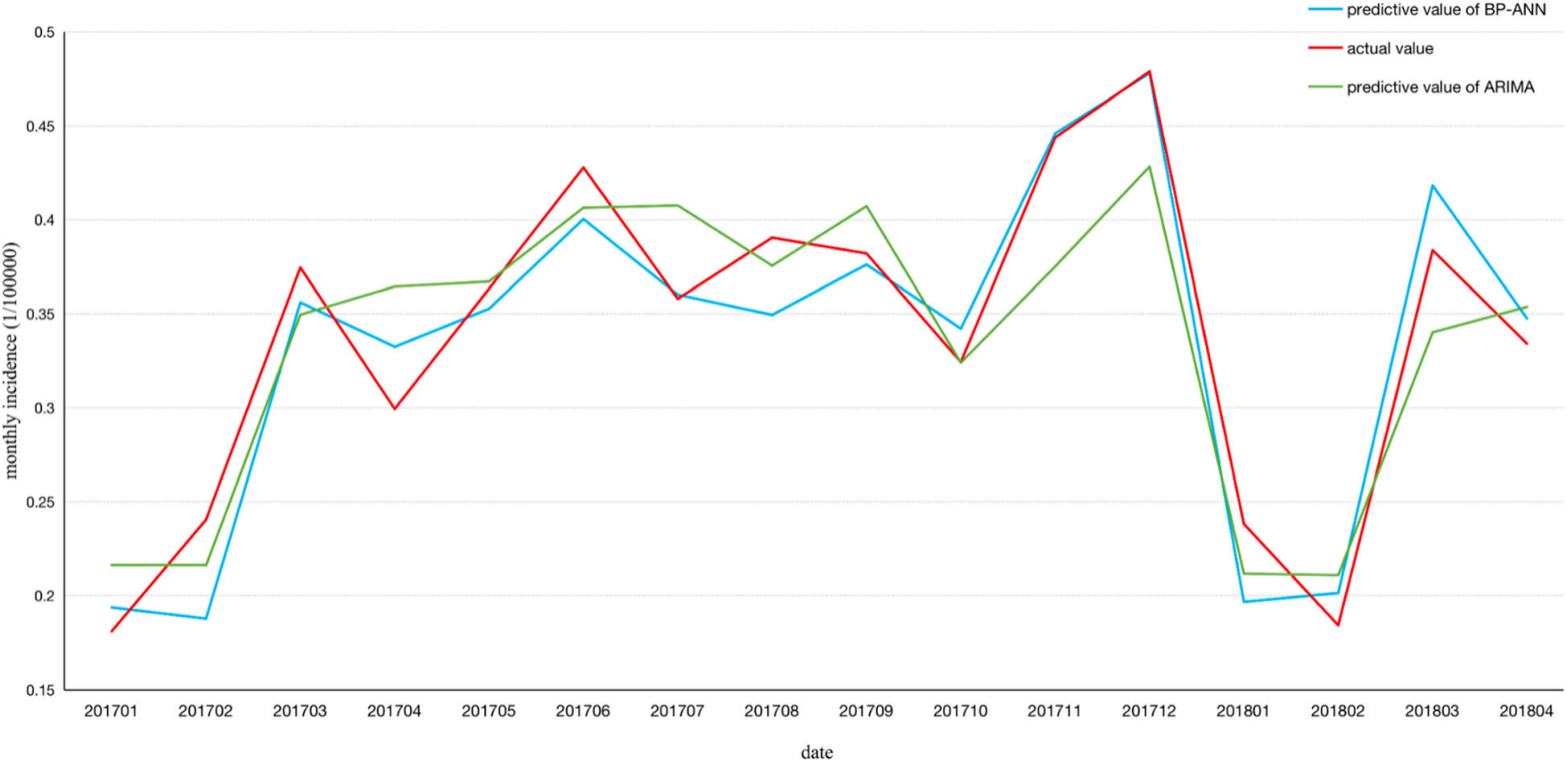
Two kinds of models to predict the monthly incidence of AIDS from January 2017 to April 2018 compared with the actual monthly incidence

In Fig. 8, the BP-ANN model had a fit value closer to the true value compared with the ARIMA model. Both prediction methods could be adopted to predict the incidence of AIDS in China. In terms of prediction accuracy, the BP-ANN model would be more suitable. The BP-ANNmodel could better improve forecasting duration than the ARIMA model. In this study, both methods just took into account the temporal variations of time series. However, the BP-ANN model was a nonlinear model, whose prediction accuracy could be enhanced by adjusting more dimensional inputs and development space was larger than that of the ARIMA model.

## Discussion

Monitoring the prevalence of infectious illnesses is of great importance for conventional health education. The prediction of anticipated AIDS cases will not only detect outburst conditions or report the possibility of outburst cases, but also help decision-makers to know about possible future change trends and past and present data [33].

Both ARIMA and BP-ANN models were based on the time series data prediction method with which the time series was extrapolated to the future through special development principles. In the model, morbidity could be predicted as special risk factors were not involved. Without complex transformations or additional alternative variables, auto-correlation, seasonal variations and secular change trends in the ARIMA model could be simply managed through seasonal functions, moving average, auto-regression and difference. As long as the suitable model was established, it would be possible to predict anticipated cases at a given time interval in the future [34].

Both models were capable of predicting the expected cases of AIDS. It was seen that both ARIMA and BP-ANN models could be used to predict the monthly incidence of HIV/AIDS, but the fitting and forecasting effects of the nonlinear BP-ANN model were superior to those of the traditional linear ARIMA model. First, the modeling method of the BP-ANNmodel was simpler than that of the ARIMA model, while it was unnecessary to set up a complicated mathematical model or understand its mathematical structure and the correlation between variables. Second, the ANN was able to compute and deal with data spontaneously through a number of simple units. It was much better to fulfill the works that were involved with pattern recognition. The professional idea was compared with traditional statistics to significantly improve the precision accuracy in neural networks. The ARIMA model might be more suitable for making short-term forecast analysis because of a gradual decline in its long-term prediction effect. As a whole, the nonlinear BP-ANN model forecasting the morbidity of AIDS in China was the most appropriate way for complicated dynamic and nonlinear systems [35]. Therefore, multi-dimensional inputs in the BP neural network would be gradually improved to find out the best model and accurately make predictions. It will be very promising in future [36].

## Conclusions

In summary, an agreement was further reached that the BP-ANN model was a suitable way to monitor and predict the change trend and morbidity of AIDS in China. According to the prediction results, more health investments would be made during outburst periods while fewer investments would be made during low-risk periods, which thus improved intervention effect and source scheduling.

### Limitations

Several limitations still exist in this study. First of all, time series analysis was carried out without considering the factors affecting the incidence of AIDS, such as production methods, social environment, epidemic variations and humanities.

Secondly, the research objects were required to remain relatively constant in prediction models during the whole process. Meanwhile, diversified infection channels and disease prevalence would be generated for a variety of people under distinct living conditions. In local places, it was necessary to re-learn and train prediction according to local conditions.

Thirdly, the BP-ANN model under black-box testing would affect the possibility of extrapolation beyond its training information and the fulfillment of subjective initiatives by operators during the process of BP-ANN analysis.

## Data Availability

The data that support the findings of this study are available from China’s Statutory Infectious Disease Report of National Health Commission of the People’s Republic of China, but restrictions apply to the availability of these data, which were used under license for the current study, and so are not publicly available.

## Abbreviations

HIV: Human Immunodeficiency Virus
AIDS: Acquired Immune Deficiency Syndrome
UNAIDS: Joint United Nations Programme on HIV/AIDS
EPP: Estimation and Projection Package
AEM: Asian Epidemic Model
ARIMA: Auto-Regressive Integrated Moving Average
ANN: Artificial Neural Network
BP-ANN: Back Propagation Artificial Neural Network
ACF: Auto-Correlation Function
PACF: Partial Auto-Correlation Function
BIC: Bayesian Information Criterions
AI: Artificial Intelligence
MAPE: Mean Absolute Percentage Error
MAE: Mean Absolute Error
MSE: Mean Square Error
